# Dosimetric impact of baseline shift plan in a magnetic resonance linear accelerator according to target motion and plan characteristics

**DOI:** 10.1002/acm2.70861

**Published:** 2026-10-20

**Authors:** Shohei Tanaka, Noriyuki Kadoya, Yoshiyuki Takahashi, Seiya Koga, Yoshiyuki Katsuta, Kazuhiro Arai, Taichi Hoshino, Yushan Xiao, Wingyi Lee, Noriyoshi Takahashi, Rei Umezawa, Keiichi Jingu

**Affiliations:** ^1^ Department of Radiation Oncology Tohoku University Graduate School of Medicine Sendai Japan

**Keywords:** Baseline shift, CMM, MR‐Linac, radiotherapy

## Abstract

**Purpose:**

In this study, the dosimetric effect of baseline shift (BLS) in a magnetic resonance linear accelerator was investigated and compared with adapt‐to‐position (ATP), adapt‐to‐shape (ATS), and an uncorrected plan without adaptation.

**Methods:**

In a simulation dataset consisting of 30 patients (10 prostate, 10 liver, 10 pancreatic), all structures were rigidly shifted by 1, 3, 5, and 10 mm separately in the superior–inferior and posterior directions, and four plans (ATS reoptimization, ATP reoptimization, BLS using the segment aperture morphing algorithm, and an uncorrected plan without adaptation) were generated for each shift condition. Planning target volume (PTV) coverage and PTV maximum dose (Dmax) were then compared. The dosimetric effects of BLS on the targets and organs at risk (OARs) under non‐rigid anatomic changes were also evaluated using a clinical dataset based on magnetic resonance imaging acquired during treatment.

**Results:**

In the simulation analysis of superior–inferior shifts, target coverage using BLS was comparable to that using ATS and ATP across all disease sites and improved compared with that using the uncorrected plan. For posterior shifts, target coverage remained comparable to that using ATS and ATP; however, BLS increased the PTV Dmax at all posterior shift magnitudes (1–10 mm) in prostate and pancreatic cases, with limited increases in liver cases. In the clinical dataset, OAR doses upon left–right or anterior–posterior displacement were greater using BLS than using ATP, ATS, and uncorrected plans in the prostate and pancreatic cases, with limited increases in the liver cases.

**Conclusions:**

The dosimetric impact of BLS relies on motion direction and plan characteristics. BLS was stable for superior–inferior shifts, whereas posterior shifts may increase PTV hotspots and OAR doses. Therefore, clinical application of BLS should consider motion direction and plan design rather than applying a uniform strategy.

## 1 INTRODUCTION

In recent years, magnetic resonance imaging (MRI) has been integrated with linear accelerators (Linac), leading to the development of magnetic resonance Linac (MR‐Linac). MRI offers superior soft tissue contrast compared with computed tomography (CT) without the associated radiation exposure. This advantage makes MRI well‐suited for adaptive radiation therapy (ART), in which the dose distribution is modified in response to anatomical changes that occur over the course of treatment. Therefore, MR‐guided ART (MRgART) has been actively investigated and is increasingly used in clinical practice. ^1^, ^2^, ^3^, ^4^, ^5^

The Elekta (Stockholm, Sweden) Unity MR‐Linac provides two ART strategy options—adapt‐to‐position (ATP) and adapt‐to‐shape (ATS)—based on anatomic information collected from daily MRI.^6^, ^7^, ^8^ In ATP, the daily MRI is registered to the reference (planning) image and the resulting positional offsets are used to reoptimize the plan such that the dose distribution (“dose cloud”) is shifted to the appropriate position, whereas the isocenter remains unchanged. This reoptimization uses the original unmodified contours. Thus, the adapted plan relies on the original planning contours.^6^ Consequently, ATP can only accommodate rigid organ shifts and is fundamentally unable to account for non‐rigid anatomical deformations. ATP optimization is limited to a simplified calculation, in which multiple organs at risk (OARs) and targets are collectively adjusted using only a small number of parameters. The second strategy, ATS, recontours based on anatomical information from the daily MRI and optimizes the treatment plan accordingly.^6^ In ATS, the planning contours can be propagated to the daily MRI using either rigid or deformable image registration. The propagated structures can be manually adjusted as required to reflect the daily anatomy. Therefore, ATS is capable of accommodating non‐rigid anatomical changes. The treatment plan is subsequently optimized based on the updated contours. Individual optimization parameters are assigned to each target and OAR, enabling more complex and precise dose optimization. ATP and ATS can be repeatedly applied before irradiation, enabling the correction of target positional shifts before beam delivery.

The Unity MR‐Linac has introduced a comprehensive motion management system in addition to the foregoing adaptive strategies. One component of the system, the baseline shift (BLS), corrects for target drift by adjusting the multi‐leaf collimator (MLC) positions. When the target is displaced (for example, because of patient motion), BLS calculates the offset between the original and displaced target positions. Assuming that the target has been rigidly shifted by the offset, the MLC aperture positions are moved accordingly. This deformation of the MLC apertures is performed using the segment aperture morphing (SAM) algorithm. The dose is then recalculated without any change to the monitor units (MUs). Clinical application of BLS has been reported in prostate cancer,^9^ abdominal tumors,^10^ and vertebral lesions.^11^ Applying BLS immediately before beam delivery may provide a rapid correction—faster than plan modification with ATS or ATP—when target positional shifts are detected. Similarly, BLS may help mitigate target underdosage or excessive OAR dose if positional shifts occur during beam delivery.^10^ However, considering the relatively large leaf width (approximately 7 mm), BLS may not fully accommodate complex target displacements in the Unity MR‐Linac system. Hence, the dose distribution may become distorted, potentially leading to target underdosage or increased OAR dose.

The dosimetric benefits of ATP and ATS in MRgART have been extensively reported.^12^, ^13^, ^14^, ^15^ Conversely, systematic dosimetric evaluations of position correction strategies involving BLS—particularly those based on dose recalculation using the SAM algorithm and direct comparison with ATP and ATS—remain limited. Consequently, guidance for clinical decision‐making regarding the optimal correction strategy in the presence of target displacement remains unclear. Specifically, whether the positional shifts detected before irradiation should be handled using ATP, ATS, or BLS remains unclear. Those three strategies differ in the time and degree of plan adaptation required. ATS enables reoptimization based on updated contours, but is the most time‐consuming adaptation. ATP is faster, but its reoptimization is limited to positional correction using the original contours. BLS enables the most rapid correction immediately before beam delivery; however, it is limited to positional correction and involves only dose recalculation with fixed MUs, without reoptimization. Moreover, when a target displacement occurs during beam delivery, real‐time reoptimization with ATP or ATS is not feasible. In that situation, beam delivery could be continued without correction (“uncorrected plan”) or BLS could be applied. The dosimetric safety and validity of applying BLS during irradiation and the way in which it compares with continuing the uncorrected plan, have not been comprehensively investigated. Quantitative comparison of motion correction strategies in terms of target coverage and OAR dose in MRgART is necessary to support clinical decision‐making.

In this study, BLS, ATS, ATP, and continuation of the uncorrected plan under target displacement were compared in terms of target coverage, hotspot formation [maximum dose (Dmax)], and OAR dose metrics.

## 2 MATERIALS AND METHODS

### 2.1 Study population and equipment

This retrospective planning simulation study included patients who had completed treatment using an MR‐Linac. Thirty patients were analyzed, comprising 10 with prostate cancer (5 low‐risk and 5 intermediate‐risk), 10 with liver cancer, and 10 with pancreatic cancer. Further, prescribed doses were 36.25 Gy in five fractions, 42 Gy in three fractions, and 40 Gy in five fractions, respectively. Table S1 presents detailed information for these patients, who constituted the simulation dataset. For each patient in the simulation dataset, one arbitrarily selected treatment fraction and its corresponding MRI and treatment plan were used for analysis. A separate clinical dataset comprising patients who demonstrated the predefined target displacement patterns during treatment (intrafractional motion) was also prepared that was used to evaluate the dosimetric effect of non‐rigid anatomic changes. The clinical case analysis is described in detail in Section 2.8. Some patients in the clinical dataset were also included in the simulation dataset.

Monaco (Elekta AB, Stockholm, Sweden) was used for the treatment planning system. The MR‐Linac used in this study was equipped with a 7‐MV flattening filter‐free beam. The source‐to‐central axis distance was 143.5 cm, the bore diameter was 70 cm, and the magnetic field strength was 1.5 T. The collimator angle was set to 90°, and the maximum field size was 57.4 × 22 cm. The MLC leaf width at the isocenter plane was 7.175 mm.

This retrospective observational study did not involve additional human sampling. The institutional review board approved the study and waived the requirement for informed consent.

### 2.2 Overview of baseline shift

In actual clinical treatment, BLS acts as follows:

BLS requires predefined targets to be designated for tracking. Automatic target tracking is performed utilizing 2D cine MRI. The baseline position is the target position at the start of treatment. The current translational shift of the target relative to that baseline position is then calculated in the left–right, anterior–posterior, and superior–inferior directions. When BLS is applied, a trailing average of the calculated target shifts is transferred to the treatment planning system to reduce temporal fluctuations. Assuming that the target has been rigidly shifted by this offset, the MLC aperture positions are moved accordingly. Dose is then recalculated with MU fixed. Dose renormalization to the prescription cannot be performed. After approval, the updated plan is transferred to the treatment console, and beam delivery resumes with the remaining MU.

The BLS plans in this simulation study were generated offline in Monaco. Displacement was simulated through the rigid translation of all structures, including targets and OARs. MLC tracking employing the SAM algorithm was then applied, followed by dose recalculation with fixed MU. In this way, a plan was virtually created after applying BLS.

### 2.3 Study workflow

Figure 1 presents the workflow of the simulation analysis. All structures (including targets and OARs) were independently translated by 1, 3, 5, and 10 mm in the superior–inferior and posterior directions for each prostate, liver, and pancreatic cancer case. The structures in prostate cases were shifted inferiorly, ^16^ and those for the liver and pancreatic cases were shifted superiorly, ^17^, ^18^, ^19^ according to reported drift tendencies. Only translational motion was simulated, while rotational displacements were not considered. Superior–inferior motion parallels the direction of the MLC movement, whereas anterior–posterior motion is perpendicular to it. In this study, we analyzed superior–inferior and anterior–posterior motion to evaluate the behavior of the SAM algorithm in response to these two physically distinct motions. Left–right motion was not simulated (see Section 4, Discussion).

**FIGURE 1:**
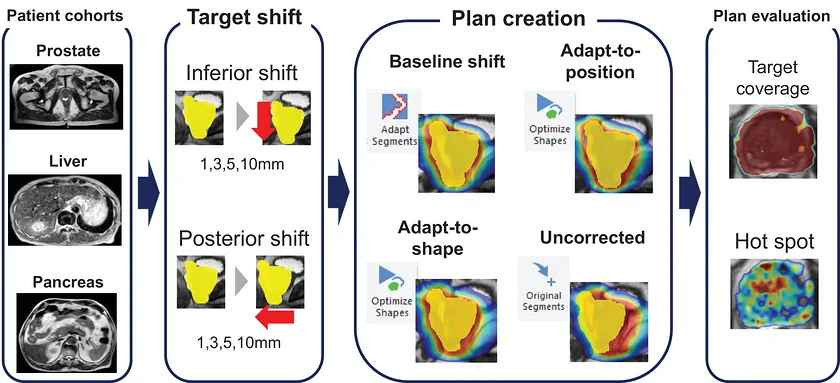
Conceptual diagram of the analysis workflow. This study included prostate, liver, and pancreatic cancer cases. To simulate target positional displacement, all structures were independently shifted by 1, 3, 5, and 10 mm in the superior–inferior and posterior directions. A dose distribution for each shift condition was generated with the application of BLS. Under the same conditions, an ATS reoptimized plan, an ATP reoptimized plan, and an uncorrected plan—recalculated without modification of monitor units or beam apertures—were generated for comparison. The BLS plan was evaluated against the ATS, ATP, and uncorrected plans in terms of target coverage and hotspot formation.

Four plans were generated for each shift condition: a BLS plan; an ATS reoptimized plan; an ATP reoptimized plan; and an uncorrected plan without modification, in which beam delivery was continued without compensating for target displacement. The impact of shift magnitude on the dosimetric characteristics of the BLS plan was assessed. The BLS plan was also compared with the ATS, ATP, and uncorrected plans in terms of target coverage.

### 2.4 Target and organ at risk delineation

Clinical MR images and structure sets originally used for clinical treatment were utilized without modification in principle.

#### 2.4.1 Prostate cancer

In all prostate cancer cases, a hydrogel spacer was placed between the prostate and rectum. The clinical target volume (CTV) included the prostate alone or the prostate plus the seminal vesicle base. The planning target volume (PTV) was generated by adding a 5‐mm margin to the CTV. The superior margin was reduced from 5 mm to 3 mm if bladder constraints were challenging to meet. The bladder was contoured as a whole organ. The urethra was contoured with a 2‐mm diameter and expanded by a 2‐mm planning risk volume (PRV) margin (total diameter: 6 mm). The rectum, small bowel, and bilateral femoral heads were delineated.

#### 2.4.2 Liver cancer

The gross tumor volume (GTV) was expanded to an internal target volume (ITV), accounting for respiratory motion. A 3‐mm margin was added to create the PTV. One case originally used a 5‐mm margin, which was modified to 3 mm for consistency. Normal liver and spinal cord were contoured. Cases with nearby OARs requiring dose reduction were excluded from this study.

#### 2.4.3 Pancreatic cancer

The GTV of the primary tumor (GTVp) was expanded to the ITV primary, considering respiratory motion, and a 3‐mm margin was added to create PTV primary (PTVp). A subclinical CTV, including major vessels with a 7‐mm margin, was defined and expanded to an ITV subclinical, followed by a 3‐mm margin to create a PTV subclinical. The clinical PTV refers to the union of PTVp and PTV subclinical; however, dosimetric evaluation focused on PTVp only, because all cases were normalized to D90 of PTVp minus PRVs. The duodenum, small bowel, large bowel, and stomach were contoured, and each was expanded by 3 mm to generate PRVs. These were combined into a single PRV structure.

### 2.5 Reference plan generation and dose calculation

In principle, the baseline plans were the treatment plans actually used for the clinical irradiation during each fraction without modification. For the present study, those plans were considered the reference plans. All clinical treatments at our institution are delivered using the ATS workflow, and when a positional shift is detected before irradiation, ATS is typically applied again for positional correction. Accordingly, all reference plans used in this study were generated using ATS. In one liver case, vascular dose constraints led to a markedly different dose distribution; these constraints were removed, and the plan was reoptimized for consistency. In one pancreatic case, the normalization was changed from D95 to D90 for consistency.

Step‐and‐shoot intensity modulated radiation therapy (IMRT) was used to create all plans. Dose normalization was D95 for prostate (PTV−rectum−bowel), D95 for liver (PTV), and D90 for pancreas (PTVp−PRVs).

The median (range) distance between the treatment isocenter and the target centroid in the reference plan was 2.6 (1.4–3.2) cm for prostate cancer, 8.4 (3.5–10.7) cm for liver cancer, and 2.4 (0.5–4.6) cm for pancreatic cancer.

All plans in this study (reference, ATS, ATP, BLS, and uncorrected) were calculated using a graphics processing unit–based Monte Carlo algorithm with a 3‐mm grid size and 1% statistical uncertainty.

Because MRI does not provide electron density information, electron densities were assigned in bulk to each structure for dose calculation. The assignment followed the method used in routine clinical practice at our institution, as described below. For liver and pancreatic cases, a representative electron density for each structure was derived from the CT numbers of the planning CT and assigned to the MRI. For prostate cancer cases, mean electron densities calculated from 20 patients were assigned to the MRI for the CTV, rectum, bladder, left femoral head, right femoral head, bone, and body structures. When rectal gas was present, those cases were excluded from the calculation of the mean value for the rectum. Because electron densities were linked to each structure, the electron density distribution was translated together with the structures during the rigid shifts in the simulation scenarios. Table S2 sets out the relative electron density assigned to each structure for each cancer type.

### 2.6 Research plan simulation

The pre‐treatment MRI on which the reference plan had been created, together with its associated structure set, was used for the research plan simulation. By duplicating the original dataset and uniformly shifting all contoured structures in the posterior and superior–inferior directions by 1, 3, 5, and 10 mm independently, eight contour datasets (radiation therapy structure sets) per case were generated.

We generated four plans for each shift condition, as follows:

1. ATS plan: Reoptimization using the “optimize shapes” algorithm with the same optimization parameters as the reference plan. This plan assumes that target position deviation is detected before irradiation, and reoptimization is performed by ATS.
2. ATP plan: Reoptimization uses the “optimize shapes” algorithm with the default ATP settings. This optimization first applies the SAM algorithm to adapt the MLC segments to the detected shift. It then performs weight and shape optimization of each segment. This plan assumes that target position deviation is detected before irradiation, and reoptimization is performed by ATP.
3. BLS plan: Generated using the SAM algorithm. Figure 2 provides a schematic illustration of MLC tracking in response to posterior and inferior target motions. Figure 2a depicts the correction of inferior target shifts through parallel inferior translation of the MLC leaves based on the magnitude of the displacement. Conversely, Figure 2b illustrates the correction of posterior target motion. As the isocenter cannot be repositioned, large displacements are compensated for by relocating the MLC leaves to an averaged position between the anterior and posterior leaf positions. This leaf‐position averaging predominantly handles posterior shifts. However, because SAM adapts the MLC based on the surface shape of the target rather than solely on the displacement of the target centroid, it might also be applied for superior–inferior shifts, depending on target shape. Following MLC tracking, the dose is recalculated while keeping the MU fixed.
4. Uncorrected plan: Recalculated without modification of the segment shapes or MUs. This plan does not represent an adaptation strategy to be selected before irradiation; rather, it serves as a baseline for quantifying the dosimetric effect of continuing beam delivery without any correction for target displacement. Clinically, it corresponds to situations in which displacement occurs during beam delivery and reoptimization cannot be performed, or in which the displacement is small and a decision is made not to apply correction.

**FIGURE 2:**
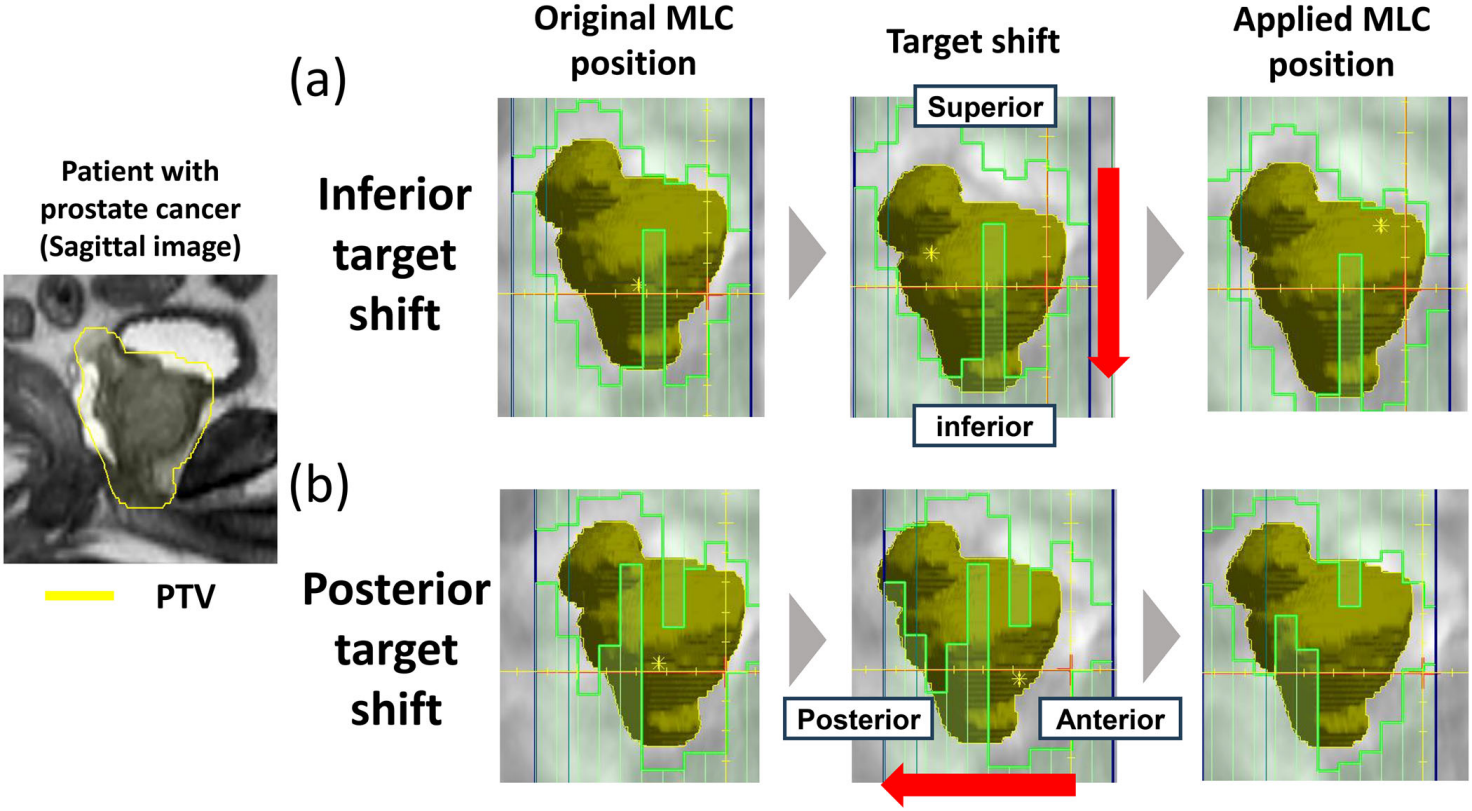
Schematic illustration of MLC tracking using the SAM algorithm. The SAM algorithm adapts the MLC to follow target displacement in the BLS plan. Both panels present a sagittal orientation as viewed from the patient's right side, corresponding to the beam's‐eye view at a gantry angle of 260 degrees. The superior, inferior, anterior, and posterior directions are indicated in the figure. (a) Schematic representation of superior–inferior (inferior) target displacement. The MLC apertures are translated in the inferior direction according to the magnitude of target shift to compensate for positional changes. Although this panel depicts parallel translation, some leaf‐position averaging might still occur depending on the target shape. (b) Schematic representation of posterior target displacement. Isocenter shifting is not available; thus, when a large displacement occurs, the MLC leaves are repositioned to the averaged location between the anterior and posterior leaf positions to compensate for target motion.

The same criteria as the reference plan were used to renormalize ATS and ATP plans. Conversely, normalization cannot be applied to the BLS and uncorrected plans in clinical practice; therefore, normalization was not performed for these plans in the present study.

### 2.7 Dosimetric evaluation

In this study's simulation analysis, all structures, including the targets, OARs, and body contour, were rigidly translated. In clinical BLS, however, only the target moves and the dose is recalculated without moving the body or OAR contours. Moreover, in actual patients, OARs undergo non‐rigid deformation rather than rigid translation. The OAR dose metrics obtained when all structures were translated might therefore not reflect clinical BLS. Accordingly, our analysis presents only target dose metrics; OAR dose metrics were evaluated from the clinical case analysis with recontouring (Section 2.8).

Target coverage (PTV−rectum−bowel and CTV) was evaluated for prostate cancer cases, PTV and ITV for liver cancer cases, and PTVp−PRVs, and GTVp for pancreatic cancer cases. The specific dose–volume histogram metrics for the targets were selected based on our institutional clinical protocol. These metrics are routinely used in clinical practice as dose constraints and plan evaluation criteria. They were adopted in this study to ensure clinical relevance. The mean values of each dosimetric parameter were calculated for each target shift magnitude (1, 3, 5, and 10 mm) in the posterior and superior–inferior directions for each plan (ATS, ATP, BLS, and uncorrected).

#### 2.7.1 Hotspot analysis

The increase in Dmax (ΔDmax) of the BLS plan relative to the reference plan was calculated for all shift magnitudes, divided into posterior shifts and superior–inferior shifts. For example, ΔDmax for posterior shifts in prostate cancer was calculated from a total of 40 data points, corresponding to four shift magnitudes (1, 3, 5, and 10 mm) across 10 patients. The Dmax values of the BLS and reference plans were expressed as a percentage of the prescribed dose. ΔDmax was defined as the Dmax of the BLS plan minus that of the reference plan.

The increase in Dmax between prostate, liver, and pancreatic cancers was compared.

Dmax was defined as

Prostate: Dmax within PTV−rectum−bowel
Liver: Dmax within PTV
Pancreas: Dmax within PTVp−PRVs

We defined the volumes used for Dmax assessment based on the structures used for dose normalization at each disease site. Since each reference plan was normalized such that prescribed doses were delivered to these target volumes, the Dmax was therefore evaluated within these volumes, where it was expected to occur.

#### 2.7.2 Exploration of factors contributing to the increase in hotspots

Spearman's rank correlation was used to evaluate associations between the Dmax increase of the BLS plan and PTV volume, PTV superior–inferior length, and plan complexity metrics, including modulation complexity score (MCS), leaf sequence variability (LSV), and aperture area variability (AAV). This analysis was conducted to identify the factors contributing to the increase in hotspots within the BLS plan. The MCS, LSV, and AAV were calculated based on the original description of their functions by McNiven et al. ^20^ Briefly, LSV quantifies the variation in MLC positions between adjacent leaf pairs, with smaller values indicating greater variability between adjacent MLC leaves. AAV quantifies how much each segment area varies relative to the maximum aperture of the field, with smaller values indicating more frequent use of smaller segment areas. MCS is the segment‐weight‐averaged product of the LSV and AAV ^20^, ^21^, ^22^. All three complexity metrics range from 0 (highly complex) to 1 (unmodulated).

This analysis was exploratory, with no multiple comparison correction being applied. Two‐sided *p*‐values of <0.05 indicated statistical significance.

#### 2.7.3 Comparison of reference plan and BLS plan complexity metrics

We performed an additional analysis to investigate changes in the complexity metrics of BLS plans relative to those of reference plans following the application of the SAM algorithm. Specifically, we aimed to determine whether the plans become more or less complex. For each prostate, liver, and pancreatic cancer case, we calculated the AAVs and LSVs for BLS plans with 3 mm superior–inferior and posterior shift conditions. We then compared these with the corresponding values in the reference plans. We selected a 3 mm shift for this analysis as it represents a clinically relevant displacement magnitude that may occur during intrafractional motion in abdominal and pelvic radiotherapy. ^16^, ^17^

Statistical significance was assessed using two‐sided *p*‐values, and *p*‐values <0.05 were considered statistically significant.

### 2.8 Dosimetric evaluation of BLS plans using clinical cases

The rigid shift applied in our simulation analysis does not reflect the non‐rigid deformation of OARs that occurs in clinical practice. To evaluate the true dosimetric effect of BLS on targets and OARs alike, we performed an additional analysis using a separate clinical dataset of treatment fractions in which real intrafractional target displacement occurred based on MRI acquired during those treatment sessions. They captured the non‐rigid anatomic changes caused by the motion.

In our analysis, we included 3D pre‐treatment, position verification, and post‐treatment MR images. Treatment fractions in which there was substantial target displacement and motion correction were also included. The two MR images acquired within each treatment fraction were extracted as pairs: the pre‐treatment MRI (hereafter, “first MRI”) and the position verification or post‐treatment MRI (hereafter, “second MRI”). The clinically used plan created on the first MRI acted as the reference plan against which the BLS plans were generated on the second MRI. This approach assumes a scenario in which the second MRI reflects the target displacement during or before irradiation, with BLS applied in response to this target displacement. We contoured the targets and OARs on the position verification and post‐treatment MR images by propagating the clinically used structures from the pre‐treatment MRI using deformable image registration, followed by manual correction.

In the clinical analysis, as in the simulation, target displacements were classified into two patterns to separately evaluate BLS behavior for superior–inferior motion (pattern 1) and for motion perpendicular to it: left–right and anterior–posterior (pattern 2).

The specific pattern definitions for prostate and pancreatic cancer cases were as follows:

- Pattern 1: superior–inferior displacement of ≥3 mm, with left–right and anterior–posterior displacements both being <1 mm.
- Pattern 2: left–right or anterior–posterior displacement of ≥3 mm (with the other direction being <1 mm), and superior–inferior displacement being <1 mm.

For liver cancer cases, the pattern definitions were as follows:

- Pattern 1: superior–inferior displacement of ≥3 mm, with left–right and anterior–posterior displacements both being <1 mm.
- Pattern 2: superior–inferior displacement of ≥3 mm, with left–right or anterior–posterior displacement being ≥3 mm (with the other direction being <1 mm)

The definition of pattern 2 for liver cancer took into account the lack of cases in which a displacement of ≥3 mm occurred exclusively in the left–right or anterior–posterior direction.

The causes of the clinically observed target displacements were, in prostate cancer cases, patient motion and contraction or relaxation of the gluteal muscles; in liver cancer cases, target drift and slight patient motion; and in pancreatic cancer cases, target drift and body motion from arching of the lumbar spine.

The clinical dataset comprised one treatment fraction from each of five different patients for each cancer type and pattern, resulting in a total of 30 treatment fractions [3 cancer types × 2 patterns × 5 fractions (Table S3)]. For each pattern, three of the five patients were also part of the simulation dataset; the remaining two patients were not included in the simulation dataset. Some patients were included in both pattern 1 and pattern 2 through different fractions. Of the liver cancer cases added to the clinical dataset, one had a vessel within the PTV. As in the simulation analysis, the vascular constraint was excluded from the optimization and the remaining clinical optimization parameters were used unchanged.

BLS, ATS, ATP, and uncorrected plans were generated for all cases, and the changes in dose constraints from the reference plan were compared for the four plans. Those plans corresponded to the plans in the simulation analysis (Section 2.6, Research Plan Simulation), but were generated on clinical MRI pairs with deformed anatomy. The generation procedures therefore differed slightly from those in the simulation. These were the specific procedures:

1. ATS plans: These plans were generated by reoptimization using the corrected contours on the second MRI, with the same optimization parameters as in the reference plan. In ATS, both the optimization and the dose calculation were based on the corrected contours on the second MRI.
2. ATP plans: These plans were generated in accordance with the clinical ATP workflow. Based on the target displacement measured between the first and second MRI, optimization was performed on the first MRI using the contours from the first MRI. However, the anatomy actually irradiated corresponded to the corrected contours on the second MRI. Therefore, the generated ATP plans were recalculated on the second MRI with the corrected contours, with the MUs and segment shapes kept fixed. The final dose metrics for the targets and OARs were obtained from the latter recalculation.
3. BLS plans: These plans were generated in accordance with the clinical BLS workflow. First, only the target contour was rigidly translated on the first MRI according to the target shift measured between the first and second MRIs; the body and OAR contours were left unchanged. Next, the SAM algorithm was applied to adjust the MLC positions, and the dose was calculated with the MUs kept fixed. That plan was then recalculated on the second MRI with the corrected contours, with the MUs and segment shapes kept fixed. The final dose metrics for the targets and OARs were then obtained from the latter recalculation.
4. Uncorrected plans: These plans were generated by recalculating the reference plan on the second MRI with the corrected contours, but without any modification of the MUs or segment shapes, thus assuming a scenario in which beam delivery was continued without any correction for anatomic deformation.

The dose calculation parameters and electron density assignments in this analysis were identical to those described in Section 2.5.

## 3 RESULTS

For the simulation analysis, Figure 3 illustrates the target dose metrics under superior–inferior (Figure 3a) and posterior (Figure 3b) shifts for the reference, ATP, ATS, uncorrected, and BLS plans. In prostate cancer cases, the uncorrected plan for inferior shifts did not compensate for target displacement (Figure 3a), leading to a reduction in PTV−rectum−bowel D98%. Conversely, the BLS plan maintained PTV−rectum−bowel D98% at levels comparable to the reference, ATP, and ATS plans. The BLS plan for posterior shifts maintained D98% target coverage comparable to that with ATP and ATS (Figure 3b). However, the PTV−rectum−bowel Dmax was higher in the BLS plan than in the ATP, ATS, and uncorrected plans across all shift magnitudes. This parameter peaked at 3–5 mm shifts.

**FIGURE 3:**
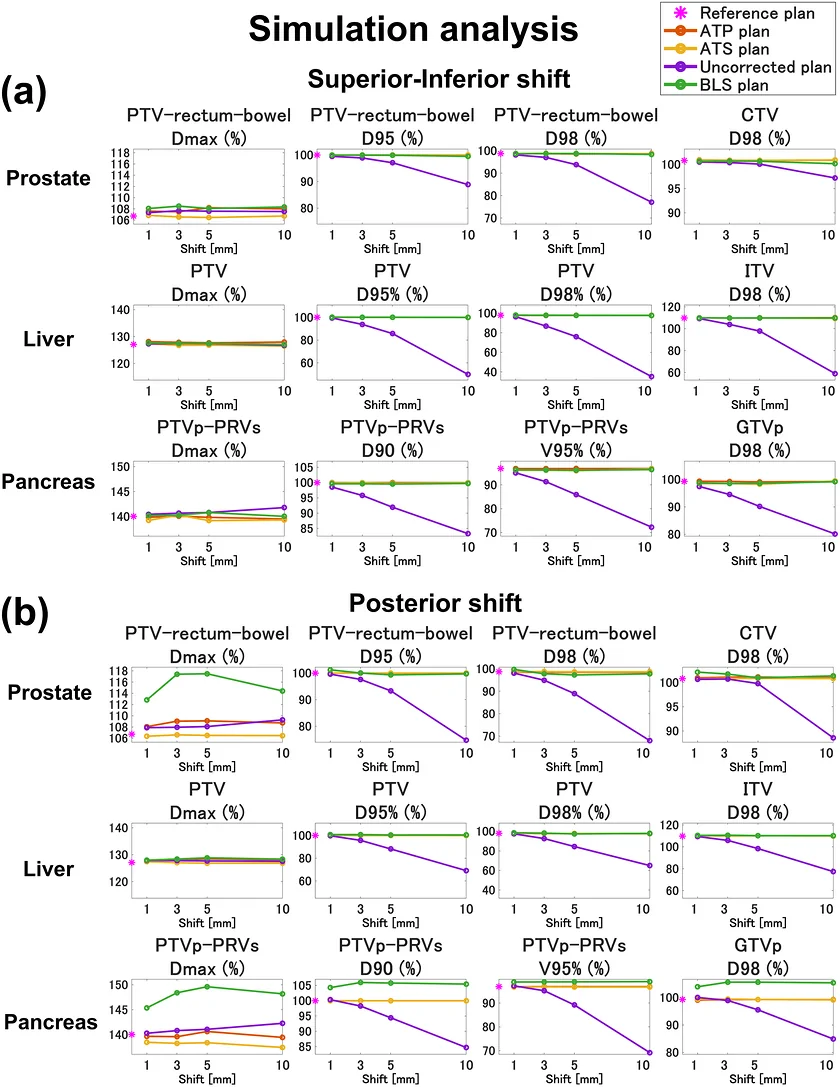
Relationship between shift magnitude and target dose metrics in the simulation analysis. Target dose metrics as a function of shift magnitude are shown for (a) superior–inferior and (b) posterior shifts. In each panel, the rows correspond to prostate (top), liver (middle), and pancreatic (bottom) cancer cases. The horizontal axis represents shift magnitude (mm), and the vertical axis represents the corresponding dose–volume metric. Each plot includes the reference plan (no shift), ATP plan, ATS plan, uncorrected plan, and BLS plan.

In liver cancer cases, the PTV D98% and Dmax for both superior (Figure 3a) and posterior (Figure 3b) shifts were maintained in the BLS plan across all shift magnitudes and were comparable to those with ATP and ATS.

In pancreatic cancer cases, the PTVp−PRVs D90% for superior shifts (Figure 3a) was maintained in the BLS plan across all shift magnitudes. Conversely, for posterior shifts (Figure 3b), the BLS plan had an increased PTVp−PRVs Dmax compared with those for ATP and ATS. The increase was most pronounced at 5 mm shifts.

Figure 4 illustrates representative dose distributions for each disease site. The BLS plan for prostate cancer (inferior shift) and liver and pancreatic cancer (superior shift) demonstrated minimal changes in isodose distribution compared with the reference plan. Conversely, expansion of high‐dose regions within the PTV for posterior shifts in prostate and pancreatic cancer was observed in the BLS plan, accompanied by distortion of isodose lines. In these cases, high‐dose regions extended closer to adjacent OARs.

**FIGURE 4:**
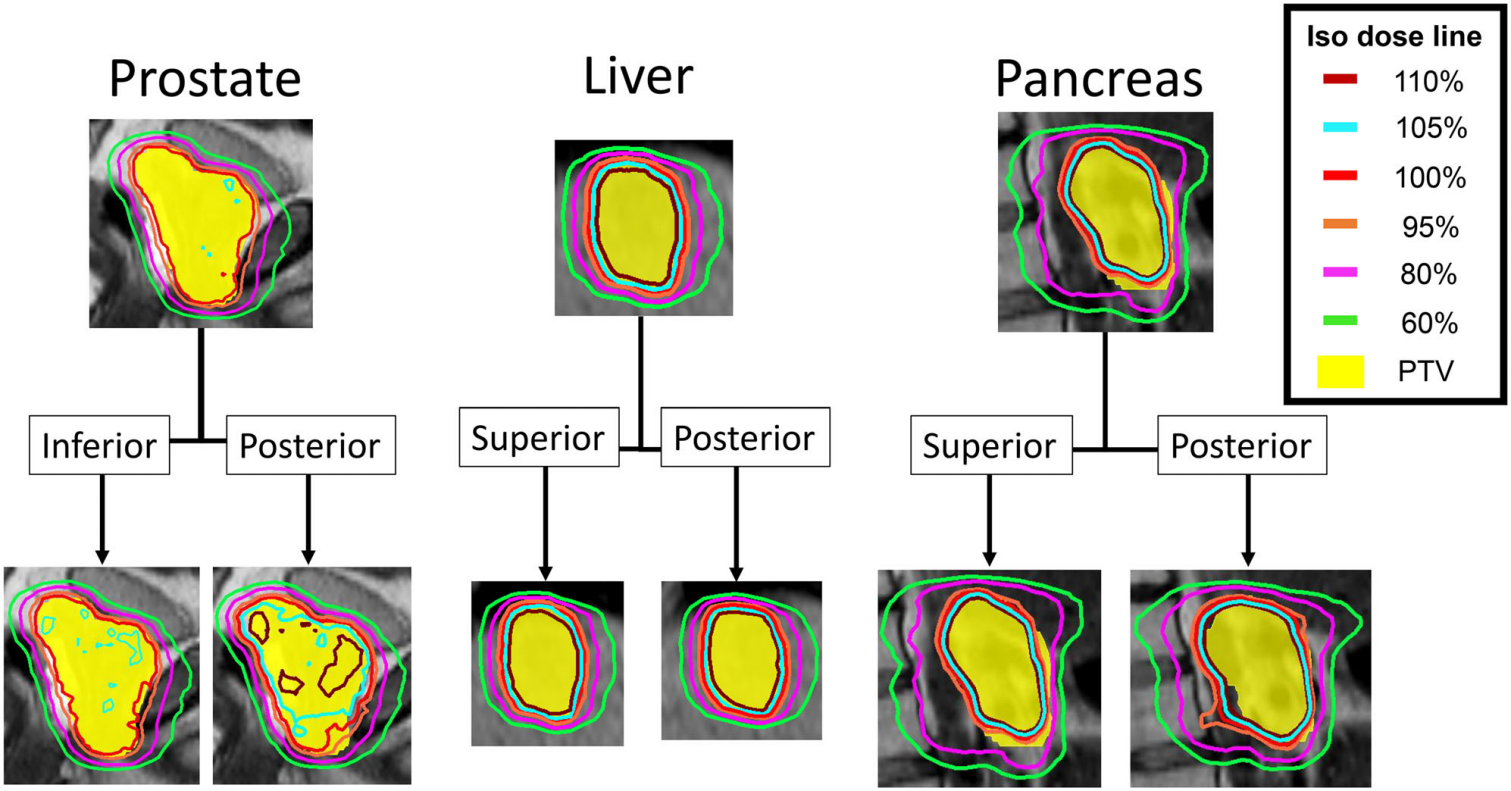
Representative dose distributions after BLS application in each disease site. Representative cases of prostate, liver, and pancreatic cancer are demonstrated under superior–inferior (inferior) and posterior shift conditions after BLS application. The upper panels display the base plan (no shift), and the lower panels demonstrate the dose distributions after BLS application for each shift direction. Isodose lines and the PTV (yellow shaded structure) are presented. Expansion of high‐dose regions within the PTV and alterations in isodose distribution were observed in posterior shifts for prostate and pancreatic cancer cases.

Figure 5 reveals the increase in Dmax (ΔDmax) for the BLS plans relative to the reference plans across all shift magnitudes (1–10 mm), separated by superior–inferior shifts (Figure 5a) and posterior shifts (Figure 5b). Dmax increases for superior–inferior shifts were generally within 5% for all disease sites. Dmax increases for posterior shifts remained within 5% for liver cancer, whereas larger increases were observed for prostate and pancreatic cancer. The maximum increase reached approximately 25% in prostate cancer.

**FIGURE 5:**
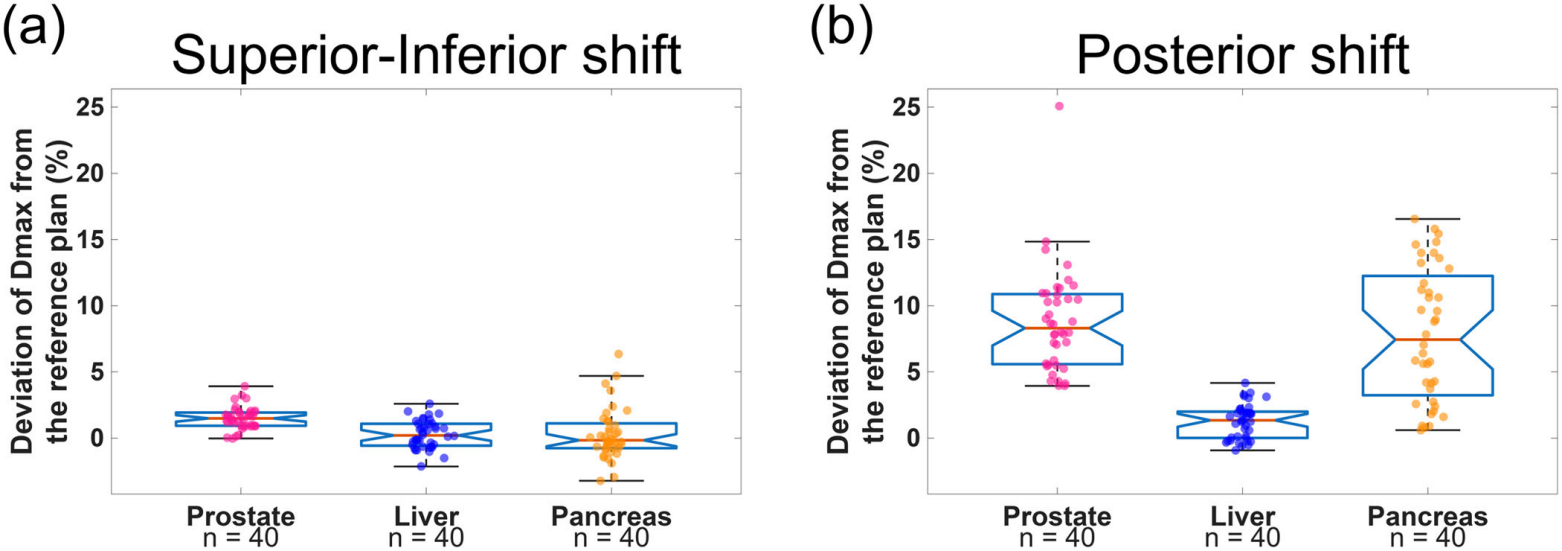
Increase in planning target volume maximum dose (ΔDmax) of the BLS plan across disease sites. The ΔDmax of the BLS plan relative to the reference plan is demonstrated for all shift magnitudes (1–10 mm), separated into (a) superior–inferior and (b) posterior shifts. ΔDmax was calculated as the Dmax of the BLS plan minus that of the reference plan, with both values expressed as a percentage of the prescribed dose. Each plot includes results for prostate, liver, and pancreatic cancer cases. The blue box plots represent the interquartile ranges, and the central red lines indicate the medians. Whiskers extend to the most extreme data points within 1.5 times the interquartile ranges. Individual data points are overlaid as colored dots (n = 40 per disease site). The increase in Dmax for superior–inferior shifts remained within approximately 5% across all disease sites. Conversely, Dmax increases for posterior shifts remained within 5% for liver cancer cases, whereas greater increases were observed in prostate and pancreatic cancer cases.

Table 1 summarizes the correlation analysis between Dmax increase and potential predictive factors. Significant positive correlations were observed between PTV volume and Dmax increase for 1‐mm and 10‐mm posterior shifts in prostate cancer and for 5‐mm posterior shifts in pancreatic cancer. PTV superior–inferior length in prostate cancer showed significant positive correlations with Dmax increase for all posterior shift magnitudes. No significant correlations were observed between plan complexity metrics (MCS, LSV, and AAV) and Dmax increase.

**TABLE 1:** Correlation between percentage increase in Dmax of the BLS plan relative to the reference plan and geometric and plan complexity factors.

Factors	Shift direction	Shift magnitude (mm)	Prostate		Liver		Pancreas	
			Spearman's correlation coefficient	*p* value	Spearman's correlation coefficient	*p* value	Spearman's correlation coefficient	*p* value
PTV volume	Superior–Inferior shift	1	0.012	0.973	−0.394	0.263	−0.055	0.892
		3	0.024	0.947	−0.382	0.279	−0.309	0.387
		5	0.055	0.880	−0.624	0.060	0.152	0.682
		10	0.098	0.789	−0.079	0.838	−0.261	0.470
	Posterior shift	1	0.799	**0.006**	−0.309	0.387	0.479	0.166
		3	0.474	0.166	0.079	0.838	0.576	0.088
		5	0.578	0.080	−0.055	0.881	0.685	**0.035**
		10	0.827	**0.003**	0.176	0.632	0.624	0.060
PTV superior–inferior length	Superior–Inferior shift	1	−0.309	0.387	−0.394	0.263	−0.152	0.682
		3	−0.200	0.584	−0.261	0.470	−0.103	0.785
		5	−0.128	0.725	−0.564	0.096	0.430	0.218
		10	−0.122	0.738	0.248	0.492	−0.152	0.682
	Posterior shift	1	0.796	**0.006**	−0.612	0.066	0.067	0.865
		3	0.709	**0.028**	−0.261	0.470	0.200	0.584
		5	0.661	**0.044**	−0.401	0.251	0.224	0.537
		10	0.721	**0.024**	0.067	0.865	0.248	0.492
MCS	Superior–Inferior shift	1	0.336	0.342	0.006	0.987	−0.081	0.824
		3	−0.404	0.247	−0.092	0.800	0.056	0.878
		5	0.126	0.729	−0.277	0.439	−0.217	0.546
		10	0.236	0.511	0.197	0.586	0.031	0.932
	Posterior shift	1	−0.429	0.216	−0.295	0.407	−0.379	0.280
		3	−0.214	0.553	0.098	0.787	−0.578	0.080
		5	−0.557	0.095	0.043	0.906	−0.491	0.150
		10	−0.330	0.351	0.375	0.285	−0.454	0.188
LSV	Superior–Inferior shift	1	−0.265	0.460	−0.067	0.865	0.128	0.724
		3	−0.597	0.068	−0.164	0.657	−0.012	0.973
		5	0.096	0.793	−0.418	0.232	−0.220	0.542
		10	0.361	0.305	0.261	0.470	−0.018	0.960
	Posterior shift	1	0.309	0.386	−0.224	0.537	0.226	0.531
		3	0.345	0.329	0.091	0.811	−0.079	0.828
		5	0.092	0.800	0.128	0.725	0.098	0.789
		10	0.178	0.622	0.345	0.331	0.262	0.464
AAV	Superior–Inferior shift	1	0.480	0.160	0.274	0.444	0.142	0.696
		3	−0.195	0.590	0.146	0.688	0.031	0.933
		5	0.122	0.737	0.389	0.266	−0.031	0.933
		10	0.104	0.776	−0.085	0.815	0.074	0.839
	Posterior shift	1	−0.543	0.105	−0.134	0.713	−0.135	0.709
		3	−0.292	0.413	−0.061	0.868	−0.295	0.407
		5	−0.584	0.077	−0.201	0.577	−0.302	0.397
		10	−0.462	0.179	−0.085	0.815	−0.172	0.634

Note: Bold values indicate statistically significant correlations (p < 0.05).

Abbreviations: BLS: baseline shift, PTV: planning target volume, MCS: modulation complexity score, LSV: leaf sequence variability, AAV: aperture area variability.

Table 2 shows the AAV and LSV of the BLS and reference plans under 3 mm superior–inferior and posterior shift conditions. For posterior shifts, the AAV was significantly higher in the BLS than in the reference plans for prostate and pancreatic cancer cases. The LSV was significantly higher in the BLS than in the reference plans across all cancer sites. For superior–inferior shifts, we found no significant difference in the AAVs between the BLS and reference plans. However, the LSV tended to be higher in the BLS than in the reference plans.

**TABLE 2:** Comparison of AAV and LSV between BLS plan (3mm posterior shift, and superior‐inferior shift) and the reference plan.

		Reference Plan	BLS plan (3mm posterior shift)	BLS plan (3mm superior‐inferior shift)	p value (Reference plan vs. BLS plan [3mm posterior shift])	p value (Reference plan vs. BLS plan [3mm superior‐inferior shift])
AAV (mean ± SD)	Prostate	0.50 ± 0.06	0.54 ± 0.06	0.49 ± 0.05	**0.002**	0.203
	Liver	0.70 ± 0.10	0.71 ± 0.10	0.70 ± 0.11	0.084	0.719
	Pancreas	0.37 ± 0.08	0.41 ± 0.07	0.37 ± 0.08	**0.002**	1.000
LSV (mean ± SD)	Prostate	0.49 ± 0.03	0.56 ± 0.02	0.51 ± 0.02	**0.002**	0.088
	Liver	0.31 ± 0.07	0.42 ± 0.06	0.33 ± 0.07	**0.002**	**0.047**
	Pancreas	0.47 ± 0.04	0.56 ± 0.03	0.49 ± 0.04	**0.002**	**0.002**

Note: Bold values indicate statistically significant correlations (p < 0.05).

Abbreviations: BLS: baseline shift, AAV: aperture area variability, SD: standard deviation, LSV: leaf sequence variability.

In the clinical case analysis, Figures 6, 7, 8 show the changes in dose constraints from the reference plan for the ATP, ATS, uncorrected, and BLS plans in the prostate (Figure 6), liver (Figure 7), and pancreatic (Figure 8) cases. For superior–inferior displacement (pattern 1) in the prostate and pancreatic cancer cases, PTV Dmax was slightly higher with the BLS plan than with the ATS, ATP, and uncorrected plans. For left–right or anterior–posterior displacement (pattern 2), the PTV Dmax and OAR doses trended highest under the BLS plan among all four plans. In the prostate cancer cases, those heightened metrics were observed for the PTV−rectum−bowel Dmax as well as for the rectum, bladder, urethra PRV, and bilateral femoral head, whereas in the pancreatic cancer cases, they were observed for the PTVp−PRVs Dmax and the small bowel and small bowel PRV (Figures 6 and 8). In contrast, for liver cancer (Figure 7), the BLS plans demonstrated comparable target coverage and OAR doses to the ATS and ATP plans for both superior–inferior displacement (pattern 1) and left–right or anterior–posterior displacement (pattern 2). Figures S1 and S2 present additional dose metrics for the prostate and pancreatic cancer cases respectively.

**FIGURE 6:**
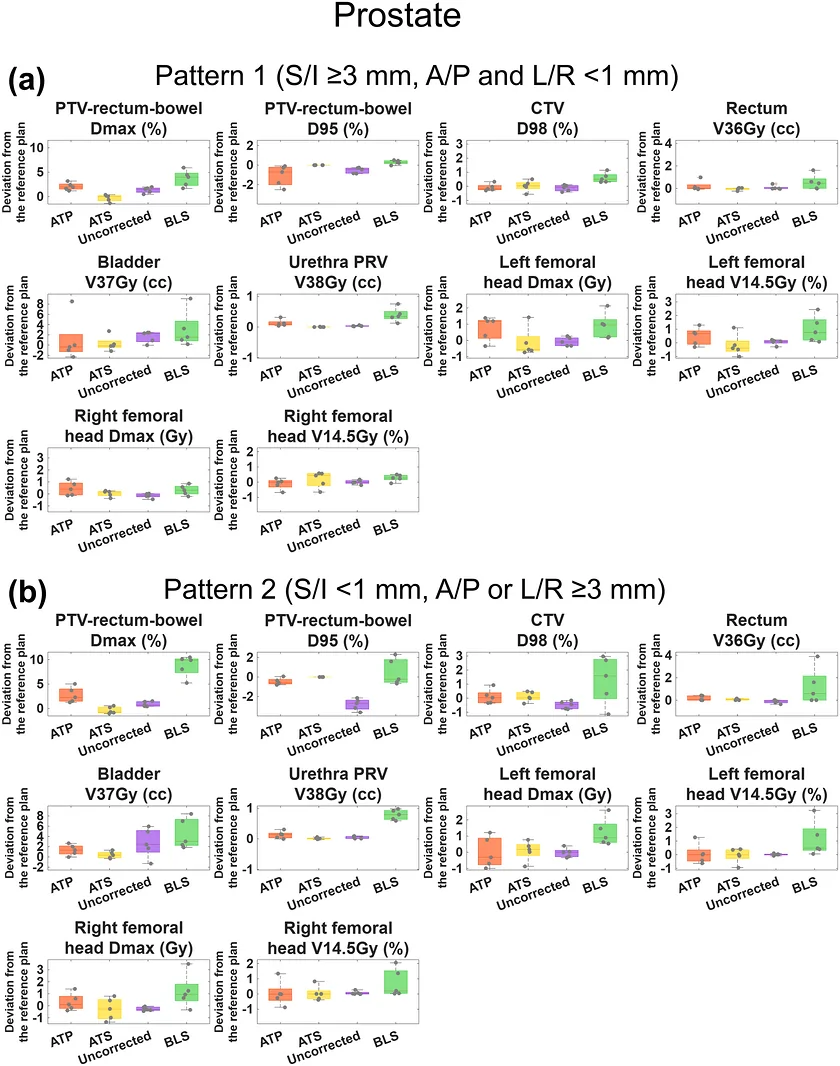
Comparison of dose metrics among ATP, ATS, uncorrected, and BLS plans in clinical prostate cancer cases. Box plots demonstrate the changes in dose metrics from the reference plan to the ATP, ATS, BLS, and uncorrected plans. The comparator plans were generated on separate MRI acquired during intrafractional motion, based on the reference plan created on the pre‐treatment 3D MRI. In each box plot, the box represents the interquartile range, the central line indicates the median, and the whiskers extend to the most extreme values within 1.5 times the interquartile range. The overlaid dots represent the individual fractions (five per pattern). ATP plans are shown in orange, ATS in yellow, uncorrected in purple, and BLS in green. (a) Pattern 1: Cases with superior–inferior (S/I) target displacement ≥3 mm and anterior–posterior (A/P) and left–right (L/R) displacement <1 mm; (b) Pattern 2: Cases with S/I target displacement <1 mm, and either A/P or L/R displacement ≥3 mm while the other is <1 mm. Additional dose metrics are presented in Figure S1.

**FIGURE 7:**
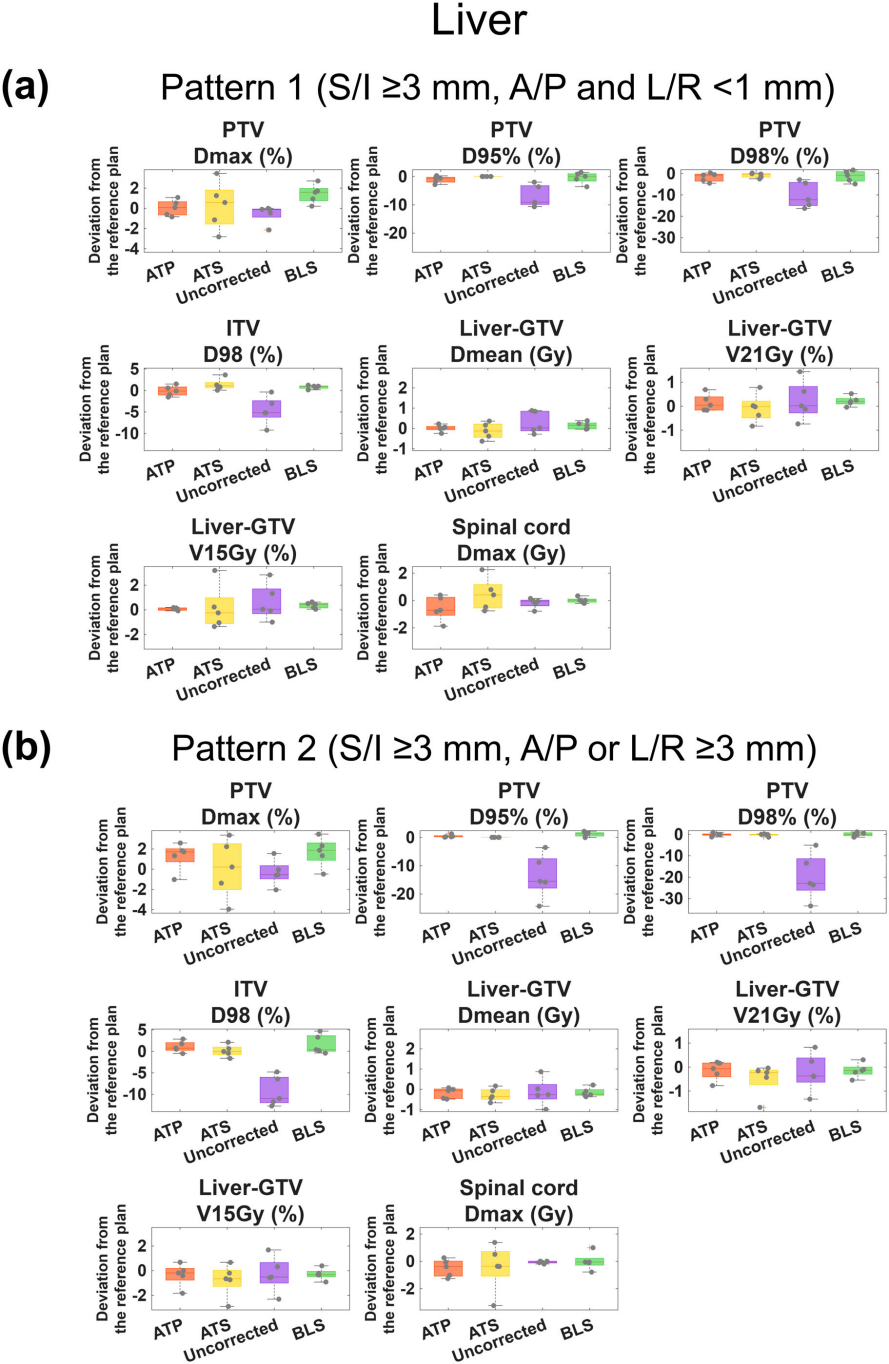
Comparison of dose metrics among ATP, ATS, uncorrected, and BLS plans in clinical liver cancer cases. Box plots demonstrate the changes in dose metrics from the reference plan to the ATP, ATS, BLS, and uncorrected plans. The comparator plans were generated on separate MRI acquired during intrafractional motion, based on the reference plan created on the pre‐treatment 3D MRI. In each box plot, the box represents the interquartile range, the central line indicates the median, and the whiskers extend to the most extreme values within 1.5 times the interquartile range; the overlaid dots represent the individual fractions (five per pattern). ATP plans are shown in orange, ATS in yellow, uncorrected in purple, and BLS in green. (a) Pattern 1: Cases with superior–inferior (S/I) target displacement ≥3 mm and anterior–posterior (A/P) and left–right (L/R) displacement <1 mm; (b) Pattern 2: Cases with S/I target displacement ≥3 mm and either A/P or L/R displacement ≥3 mm while the other is <1 mm.

**FIGURE 8:**
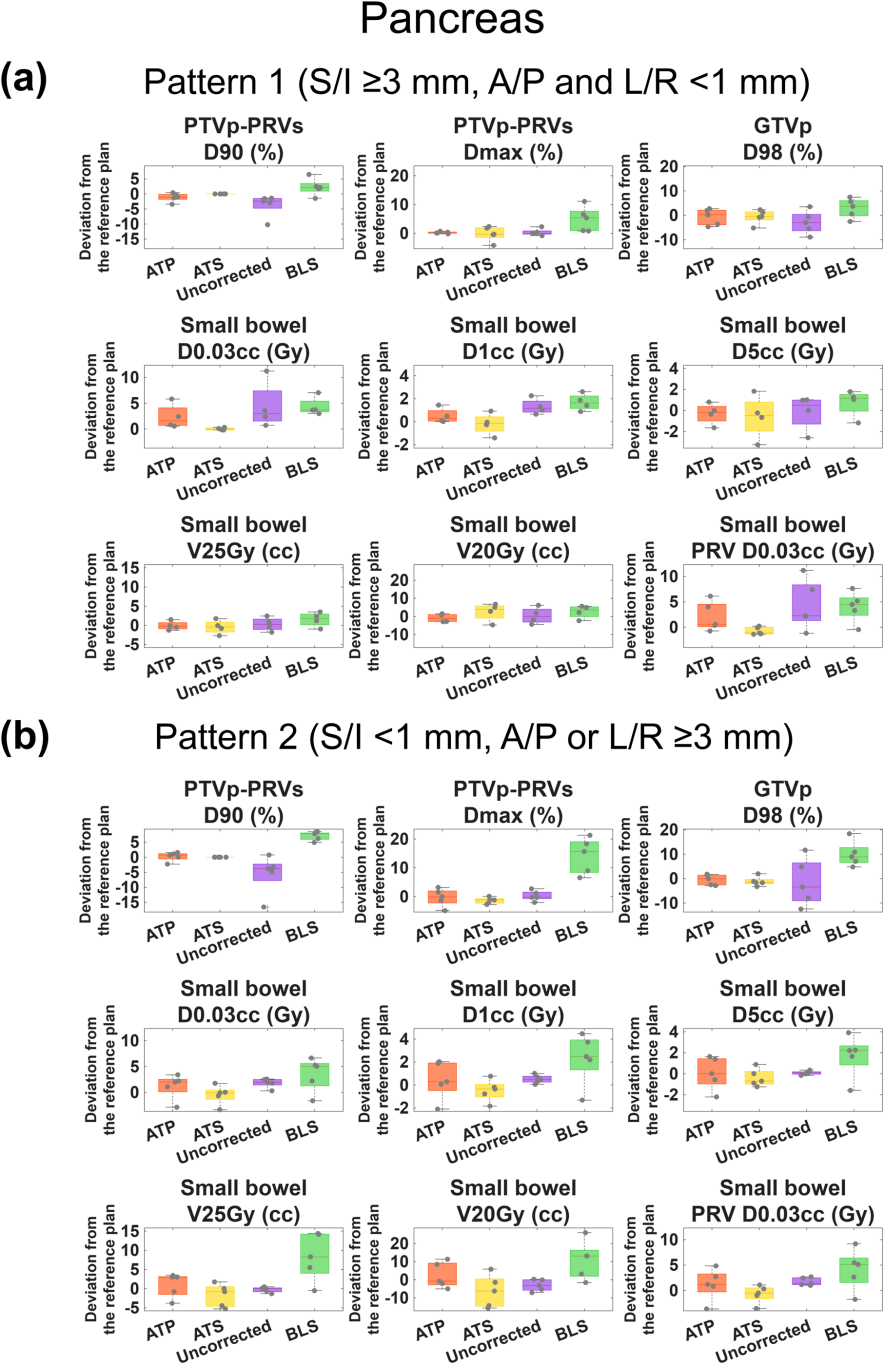
Comparison of dose metrics among ATP, ATS, uncorrected, and BLS plans in clinical pancreatic cancer cases. Box plots demonstrate the changes in dose metrics from the reference plan to the ATP, ATS, BLS, and uncorrected plans. The comparator plans were generated on separate MRI acquired during intrafractional motion, based on the reference plan created on the pre‐treatment 3D MRI. In each box plot, the box represents the interquartile range, the central line indicates the median, and the whiskers extend to the most extreme values within 1.5 times the interquartile range; the overlaid dots represent the individual fractions (five per pattern). ATP plans are shown in orange, ATS in yellow, uncorrected in purple, and BLS in green. (a) Pattern 1: Cases with superior–inferior (S/I) target displacement ≥3 mm and anterior–posterior (A/P) and left–right (L/R) displacement <1 mm; (b) Pattern 2: Cases with S/I target displacement <1 mm, and either A/P or L/R displacement ≥3 mm while the other is <1 mm. Additional dose metrics are presented in Figure S2.

## 4 DISCUSSION

In this study, we compared BLS plans with ATS, ATP, and uncorrected plans to investigate the dosimetric impact of target displacement in an MR‐Linac system. A clear direction‐dependent effect was observed. Superior–inferior shifts led to minimal perturbation of the dose distribution, whereas posterior shifts produced more pronounced dosimetric changes (Figures 3 and 4). Target motion for superior–inferior shifts largely coincides with the direction of MLC leaf travel (Figure 2a). Geometric compensation can therefore be achieved by translating individual leaves according to the measured shift, leading to relatively stable dose distributions, although some leaf‐position averaging might still occur depending on the target shape.

Left–right target shifts were not included in this analysis because the results were expected to be similar to those observed for anterior–posterior shifts. Like anterior–posterior motion, left–right motion is perpendicular to the direction of MLC leaf travel. Thus, in treatment plans with coplanar beams arranged at various angles, the SAM algorithm would be expected to exhibit similar behavior for both directions.

Grimbergen et al. reported improved agreement with planned dose distributions when gating was combined with BLS in abdominal tumors compared with continued beam delivery without gating.^10^ However, their analysis was conducted based on accumulated dose calculated from isocenter shifts rather than dose recalculation using the SAM algorithm. Conversely, studies incorporating SAM‐based dose recalculation have compared BLS with uncorrected plans. Ding et al. showed that BLS preserved target coverage but increased spinal cord dose in spine stereotactic radiation therapy.^11^ Van den Dobbelsteen et al. reported that BLS improved PTV coverage in prostate cancer while increasing rectal and bladder doses.^23^ With respect to our comparison of uncorrected and BLS plans, the results are broadly consistent with those previous studies, which compared BLS with uncorrected plans. Furthermore, to our knowledge, our study is the first to directly compare BLS with ATP and ATS, and to evaluate its dosimetric effect across shift magnitudes, directions, and disease sites.

The impact of BLS during posterior shifts varied among disease sites. Increases in PTV Dmax were limited even for posterior shifts in liver cancer, whereas prostate and pancreatic cancers demonstrated a more pronounced increase in hotspots (Figure 5). The selected liver cases did not include nearby OARs requiring strict dose sparing, and prescription isodose lines were approximately 80%, enabling dose inhomogeneity within the PTV. Hence, MLC leaves did not need to extend deeply into the PTV, and the plans resembled relatively simple, three‐dimensional conformal radiation therapy (3DCRT)‐like IMRT configurations. In such plans, only minor MLC adjustments were needed with BLS, and the resulting dosimetric changes were limited. Conversely, prostate plans emphasized dose homogeneity within the PTV, with MLC leaves extending more deeply into the target. SAM‐based leaf reconstruction was constrained by leaf width and orientation when posterior shifts occurred, leading to dose redistribution and increased PTV Dmax. Similarly, pancreatic plans were inherently complex due to the proximity of critical OARs, thereby requiring steep dose gradients within the PTV. These plans were presumed to be more susceptible to substantial dosimetric changes with BLS application during posterior shifts. These findings indicate that the dosimetric safety of BLS relies not only on motion direction but also on plan design and intrinsic dose characteristics.

Table S2 presents the relative electron densities assigned to each structure. The relative electron densities of the target and surrounding tissues were comparable for the prostate cancer cases (CTV, 1.046; rectum, 1.039; bladder, 1.025) and liver cancer cases (ITV, 1.041; normal liver, 1.059), and all were close to that of soft tissue and relatively homogeneous. Nevertheless, the increase in PTV Dmax with BLS under posterior shifts was greater in the prostate cancer cases than in the liver cancer cases (Figures 3b and 5b). That observation suggests that the differences between disease sites might be attributable mainly to plan characteristics rather than tissue homogeneity.

For left–right or anterior–posterior displacement (pattern 2) of the clinical case analysis, BLS increased the OAR doses relative to the reference plan in the prostate and pancreatic cancer cases. In prostate cases, metrics for the rectum, bladder, urethra PRV, and bilateral femoral head increased (Figure 6b), whereas in pancreatic cases, metrics for the small bowel and small bowel PRV increased (Figure 8b). In BLS, the SAM algorithm averages the positions of adjacent MLC leaves to form the treatment aperture. This smooths out any MLC leaves that originally protruded into the PTV. Consequently, the treatment aperture becomes wider, and the dose is concentrated within the PTV. It then accumulates across all beam directions, presumably causing the high‐dose region to extend beyond the PTV. Supporting this hypothesis, in the simulation analysis, we found significantly higher AAV and LSV values in the BLS plans compared with the reference plans when a 3 mm posterior shift was applied in prostate and pancreatic cancer cases (Table 2). This confirmed that BLS had increased the segment aperture area and reduced the variation between adjacent leaf positions. The expansion of the dose distribution beyond the PTV resulting from the broader treatment aperture in BLS plans is likely to be the cause of the increased OAR dose following BLS application. In contrast, target coverage was maintained after BLS application in all disease sites regardless of shift direction, both in the simulation (Figure 3) and clinical (Figures 6, 7, 8) analyses. The broader treatment aperture simultaneously maintained the dose within the PTV, which likely explains why target coverage was not compromised.

In the clinical analysis, the PTV Dmax and high‐dose OAR metrics (bladder V37Gy for prostate; small bowel D0.03cc for pancreas) were slightly higher with BLS than with ATP in the prostate and pancreatic cancer cases (Figures 6a and 8a), even for the 3 mm superior–inferior shift. Unlike the purely superior–inferior displacement in the simulation, the clinical pattern 1 cases included small left–right and anterior–posterior components, and the SAM algorithm smoothed adjacent leaf positions. The LSV was higher than in the reference plan in both the prostate (reference, 0.524 ± 0.048; BLS, 0.562 ± 0.050) and pancreatic (reference, 0.438 ± 0.024; BLS, 0.512 ± 0.022) cancer cases, and the AAV also increased in the prostate cancer cases (reference, 0.462 ± 0.046; BLS, 0.486 ± 0.034). This modest aperture smoothing likely contributed to a lack of dosimetric advantage for BLS over ATP for the clinical 3 mm superior–inferior shifts.

In this study, the dosimetric impact of BLS relied on target motion direction and plan characteristics. We discuss how ART strategies incorporating BLS may be selected in clinical practice based on these findings. BLS preserved target coverage for superior–inferior shifts across the prostate, liver, and pancreatic cancer cases (Figure 3a). Moreover, for superior–inferior displacement (pattern 1) in the clinical case analysis, a slight tendency toward increased Dmax and OAR doses was observed (Figures 6a and 8a); however, the changes were small. BLS might be an effective correction strategy for superior–inferior positional shifts identified before beam delivery and its applicability might also extend to shifts occurring during delivery. Indeed, the clinical scenario analysis included displacements identified on post‐treatment MRI (reflecting motion that occurred during the fraction), and the characteristics of patient motion are unlikely to differ substantially before and during beam delivery. It should be noted, however, that the effectiveness of BLS as a real‐time correction during beam delivery was not directly evaluated in this study.

For posterior shifts, BLS is also an effective strategy in relatively simple, 3DCRT‐like plans such as liver, where its dosimetric impact was minimal (Figures 3b and 7b). In the more modulated prostate and pancreatic plans, however, PTV Dmax increased across all shift magnitudes of 1–10 mm (Figure 3b); therefore, prioritizing ATP or ATS reoptimization before beam delivery may be preferable when feasible, although these require additional time. However, since real‐time reoptimization using ATP or ATS is not possible during beam delivery, one of three strategies must be selected. These are the continuation of the uncorrected plan, the application of BLS, or the adoption of a completion plan. In the completion plan, the dose distribution for the remaining dose, excluding the dose already delivered, is reconstructed using ATP. A completion plan requires offline generation of the remaining dose distribution, re‐registration as a new reference plan, and subsequent re‐execution of the ATP process.^24^ Therefore, this approach imposes additional time demands. In the simulation analysis, even small posterior shifts (1–3 mm) increased the PTV Dmax when BLS was compared with uncorrected plans (Figure 3b). When target displacement remained within the PTV margin (5 mm for prostate and 3 mm for pancreas), CTV or GTV D98% was preserved with the uncorrected plan (Figure 3b). According to these findings, continuation of the uncorrected plan rather than applying BLS without careful consideration may be reasonable in such situations. Conversely, when displacement exceeds the PTV margin and target coverage is compromised, BLS may be considered when the risk of an increased OAR dose is clinically acceptable. However, a completion plan may be the most appropriate strategy when neither a reduction in target coverage nor an increase in OAR dose is acceptable. Since a completion plan utilizes ATP optimization, it is more likely than continuation of the uncorrected plan or BLS to maintain target coverage while reducing the OAR dose. Beyond motion direction and plan characteristics, the appropriate adaptation strategy also depends on the selected CTV–PTV margin. With a relatively large margin, target coverage is likely to be maintained under non‐rigid deformation, regardless of whether ATP, BLS, or the uncorrected plan is used. With a smaller margin, however, non‐rigid deformation might compromise coverage, increasing the need for ATS—which recontours and reoptimizes to the daily anatomy—rather than ATP or BLS, which correct only rigid shifts.

This study has several limitations. First, it was a single‐institution retrospective simulation study, and planning strategies and machine configurations reflect local clinical practice. External validation is required to identify generalizability. Second, the sample sizes were limited—10 cases per disease site in the simulation analysis and five cases per pattern in the clinical case analysis. Larger cohorts might yield more robust conclusions. Third, target displacement was modeled as a fixed translational shift persisting throughout beam delivery. Target motion is dynamic in clinical reality, and cumulative dose should be assessed by integrating the reference and BLS‐modified dose distributions over time. Such temporal dose accumulation was not considered and warrants future investigation employing dynamic motion models. Fourth, the distance between the treatment isocenter and the target centroid was not standardized between cases in this study. Since the 7 MV beam was unflattened, the distance between the treatment isocenter and the target centroid in the reference plan affected target coverage in the BLS plans. Table S4 shows the correlation between the isocenter‐to‐target‐centroid distance and the target coverage of the BLS plans. In the prostate cancer cases, those with larger isocenter‐to‐target‐centroid distances tended to show less target coverage. The mean PTV−rectum−bowel D98% of the BLS plan for the prostate cancer cases with the largest isocenter‐to‐target‐centroid distance (3.2 cm) and the smallest distance (1.4 cm) were 98.0% and 99.2%, respectively. While this difference was minor, it should be borne in mind that it was inherently included in the target coverage analysis of this study.

## 5 CONCLUSION

This study investigated the dosimetric impact of BLS in an MR‐Linac system in comparison with ATP, ATS, and uncorrected plans, considering motion direction and plan characteristics. For superior–inferior shifts, BLS maintained dose distributions that were largely stable, with only minor increases in PTV Dmax and OAR doses. Conversely, posterior shifts increased PTV hotspots in highly modulated prostate and pancreatic plans, even for small displacements, and OAR doses were also elevated in the clinical analysis. Those results indicate that BLS should not be applied uniformly. Instead, clinical decisions should integrate motion direction, displacement magnitude, and plan‐specific dose characteristics to ensure safe and appropriate adaptive management.

## AUTHOR CONTRIBUTIONS

ST mainly drafted the manuscript. NK and KJ reviewed the manuscript. ST, YT and SK performed the analysis. YK, KA, TH, YX, WL provided guidance regarding planning and MR‐Linac. NT and RU provided clinical knowledge. All authors read and approved the final manuscript.

## CONFLICT OF INTEREST STATEMENT

Dr. Keiichi Jingu has received a research grant from Elekta.

## ETHICS APPROVAL

This study was approved by the institutional ethics committee. All procedures were conducted in accordance with institutional and national ethical standards and regulations.

## Supporting information




**Supporting Information**: acm270861‐sup‐0001‐FigureS1.pdf.

**Supporting Information**: acm270861‐sup‐0002‐FigureS2.pdf.

**Supporting Information**: acm270861‐sup‐0003‐TableS1.docx.

**Supporting Information**: acm270861‐sup‐0004‐TableS2.docx.

**Supporting Information**: acm270861‐sup‐0005‐TableS3.docx.

**Supporting Information**: acm270861‐sup‐0006‐TableS4.docx

## Data Availability

Due to the restrictions of the institutional ethics board approval and patients’ consent, the raw imaging and clinical data cannot be publicly available. However, de‐identified derived data supporting the findings of this study may be shared upon reasonable request, subject to approval by the institutional ethics board.
