# Supplementary material for: Dosimetric impact of baseline shift plan in a magnetic resonance linear accelerator according to target motion and plan characteristics
Source: J Appl Clin Med Phys. 2026 Oct 20;27(11):e70861. doi: 10.1002/acm2.70861 (PMC13682616; doi:10.1002/acm2.70861)
Supplement: Supplementary file 1 [file ACM2-27-e70861-s002.pdf]

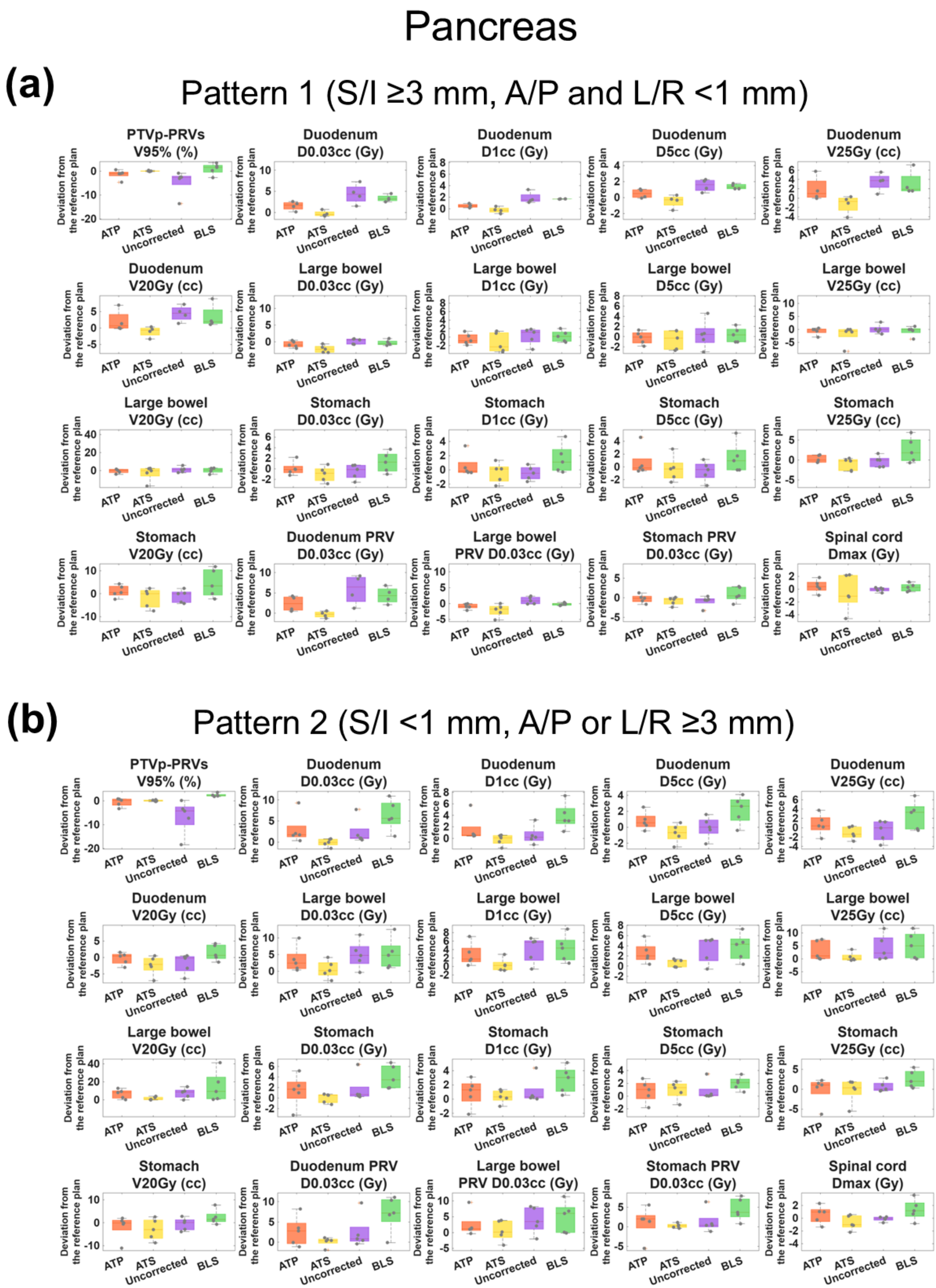

Supplementary Figure S2. Additional dose metrics for the clinical pancreatic cancer cases

For the dose metrics not included in Figure 8, box plots demonstrate the changes from the

reference plan to the ATP, ATS, BLS, and uncorrected plans. As in Figure 8, the comparator plans were generated on separate MRI acquired during intrafractional motion, based on the reference plan created on the pre-treatment 3D MRI. In each box plot, the box represents the interquartile range, the central line indicates the median, and the whiskers extend to the most extreme values within 1.5 times the interquartile range; the overlaid dots represent the individual fractions (five per pattern). ATP plans are shown in orange, ATS in yellow, uncorrected in purple, and BLS in green.

(a) Pattern 1: Cases with superior–inferior (S/I) target displacement ≥3 mm and anterior–posterior (A/P) and left–right (L/R) displacement <1 mm;

(b) Pattern 2: Cases with S/I target displacement <1 mm, and either A/P or L/R displacement ≥3 mm while the other is <1 mm.
