# Supplementary material for: Dosimetric impact of baseline shift plan in a magnetic resonance linear accelerator according to target motion and plan characteristics
Source: J Appl Clin Med Phys. 2026 Oct 20;27(11):e70861. doi: 10.1002/acm2.70861 (PMC13682616; doi:10.1002/acm2.70861)
Supplement: Supplementary file 3 [file ACM2-27-e70861-s001.docx]

**Supplementary Table S1. Patient characteristics of the simulation dataset in this study.**

| Patient Number | Age | Sex | Diagnosis | Prescribed dose | PTV volume (cm^3^) | PTV superior–inferior length (cm) |
| --- | --- | --- | --- | --- | --- | --- |
| 1 | 84 | M | Prostate cancer (low risk) | 36.25 Gy in 5 fractions | 88.1 | 5.6 |
| 2 | 69 | M | Prostate cancer (low risk) | 36.25 Gy in 5 fractions | 47.4 | 4.8 |
| 3 | 56 | M | Prostate cancer (low risk) | 36.25 Gy in 5 fractions | 71.6 | 5.9 |
| 4 | 64 | M | Prostate cancer (low risk) | 36.25 Gy in 5 fractions | 62.6 | 5.8 |
| 5 | 75 | M | Prostate cancer (low risk) | 36.25 Gy in 5 fractions | 114.0 | 7.1 |
| 6 | 80 | M | Prostate cancer (intermediate risk) | 36.25 Gy in 5 fractions | 114.0 | 7.4 |
| 7 | 69 | M | Prostate cancer (intermediate risk) | 36.25 Gy in 5 fractions | 110.5 | 7.2 |
| 8 | 70 | M | Prostate cancer (intermediate risk) | 36.25 Gy in 5 fractions | 68.9 | 5.8 |
| 9 | 72 | M | Prostate cancer (intermediate risk) | 36.25 Gy in 5 fractions | 95.7 | 7.0 |
| 10 | 83 | M | Prostate cancer (intermediate risk) | 36.25 Gy in 5 fractions | 52.1 | 5.6 |
| 11 | 86 | M | Liver cancer | 42 Gy in 3 fractions | 9.6 | 3.4 |
| 12 | 76 | M | Liver cancer | 42 Gy in 3 fractions | 33.0 | 4.5 |
| 13 | 81 | M | Liver cancer | 42 Gy in 3 fractions | 11.4 | 3.0 |
| 14 | 49 | F | Liver cancer | 42 Gy in 3 fractions | 28.7 | 4.6 |
| 15 | 91 | M | Liver cancer | 42 Gy in 3 fractions | 22.1 | 4.1 |
| 16 | 80 | F | Liver cancer | 42 Gy in 3 fractions | 11.9 | 3.2 |
| 17 | 86 | F | Liver cancer | 42 Gy in 3 fractions | 22.7 | 2.8 |
| 18 | 74 | M | Liver cancer | 42 Gy in 3 fractions | 11.7 | 3.3 |
| 19 | 75 | F | Liver cancer | 42 Gy in 3 fractions | 62.6 | 4.9 |
| 20 | 78 | M | Liver cancer | 42 Gy in 3 fractions | 12.3 | 3.5 |
| 21 | 61 | M | Pancreas cancer | 40 Gy in 5 fractions | 64.4 | 5.7 |
| 22 | 74 | F | Pancreas cancer | 40 Gy in 5 fractions | 22.3 | 4.2 |
| 23 | 55 | F | Pancreas cancer | 40 Gy in 5 fractions | 34.0 | 4.8 |
| 24 | 69 | F | Pancreas cancer | 40 Gy in 5 fractions | 20.4 | 2.6 |
| 25 | 57 | F | Pancreas cancer | 40 Gy in 5 fractions | 21.0 | 5.0 |
| 26 | 77 | M | Pancreas cancer | 40 Gy in 5 fractions | 103.4 | 5.7 |
| 27 | 76 | M | Pancreas cancer | 40 Gy in 5 fractions | 50.2 | 4.2 |
| 28 | 47 | M | Pancreas cancer | 40 Gy in 5 fractions | 71.5 | 6.8 |
| 29 | 74 | F | Pancreas cancer | 40 Gy in 5 fractions | 43.2 | 4.5 |
| 30 | 65 | M | Pancreas cancer | 40 Gy in 5 fractions | 43.0 | 5.0 |

PTV: planning target volume
