# Supplementary material for: Dosimetric impact of baseline shift plan in a magnetic resonance linear accelerator according to target motion and plan characteristics
Source: J Appl Clin Med Phys. 2026 Oct 20;27(11):e70861. doi: 10.1002/acm2.70861 (PMC13682616; doi:10.1002/acm2.70861)
Supplement: Supplementary file 4 [file ACM2-27-e70861-s004.docx]

**Supplementary Table S3. Detailed information of the clinical dataset.**

| Diagnosis | Pattern | Patient Number | Target S/I displacement (mm) | Target A/P displacement (mm) | Target L/R displacement (mm) |  |
| --- | --- | --- | --- | --- | --- | --- |
| Prostate cancer (low risk) | 1 | 1 | 4.0 | −0.2 | −0.9 |  |
| Prostate cancer (intermediate risk) | 1 | 6 | −3.0 | −0.2 | 0.5 |  |
| Prostate cancer (intermediate risk) | 1 | 9 | −3.0 | 0.0 | 0.8 |  |
| Prostate cancer (low risk) | 1 | N1 | −3.0 | 0.0 | 0.0 |  |
| Prostate cancer (intermediate risk) | 1 | N2 | −3.0 | −0.2 | 0.4 |  |
| Prostate cancer (low risk) | 2 | 2 | 0.0 | −3.9 | 0.9 |  |
| Prostate cancer (low risk) | 2 | 4 | 0.0 | 3.7 | 0.5 |  |
| Prostate cancer (intermediate risk) | 2 | 7 | 0.0 | −3.5 | 0.3 |  |
| Prostate cancer (intermediate risk) | 2 | N3 | 0.0 | −3.4 | 0.6 |  |
| Prostate cancer (intermediate risk) | 2 | N4 | 0.0 | −3.2 | 0.1 |  |
| Liver cancer | 1 | 15 | 3.0 | 0.5 | −0.4 |  |
| Liver cancer | 1 | 19 | 4.8 | −0.4 | 0.7 |  |
| Liver cancer | 1 | 20 | 3.0 | −0.9 | −0.2 |  |
| Liver cancer | 1 | N5 | 3.6 | −0.5 | −0.5 |  |
| Liver cancer | 1 | N6 | 3.6 | −0.5 | 0.7 |  |
| Liver cancer | 2 | 14 | 5.4 | −0.5 | −3.9 |  |
| Liver cancer | 2 | 18 | 3.6 | −3.4 | −0.3 |  |
| Liver cancer | 2 | 20 | 6.6 | −3.7 | −0.2 |  |
| Liver cancer | 2 | N7 | 6.0 | −3.2 | 0.7 |  |
| Liver cancer | 2 | N8 | 3.6 | −3.4 | 0.6 |  |
| Pancreas cancer | 1 | 21 | 3.0 | −0.4 | 0.5 |  |
| Pancreas cancer | 1 | 22 | 5.0 | −0.4 | −0.1 |  |
| Pancreas cancer | 1 | 29 | 3.0 | 0.3 | −0.1 |  |
| Pancreas cancer | 1 | N9 | 4.0 | 0.7 | −0.2 |  |
| Pancreas cancer | 1 | N10 | 3.6 | 0.7 | 0.7 |  |
| Pancreas cancer | 2 | 23 | 0.0 | −0.4 | 3.5 |  |
| Pancreas cancer | 2 | 26 | 0.0 | 0.4 | 3.6 |  |
| Pancreas cancer | 2 | 30 | 0.0 | 0.9 | −5.9 |  |
| Pancreas cancer | 2 | N9 | 0.0 | 3.6 | −0.1 |  |
| Pancreas cancer | 2 | N11 | 0.0 | −4.1 | 0.9 | |

Patient numbers correspond to those in Supplementary Table S1; patients labeled N1–N11 were additional patients not included in the simulation dataset. Some patients contributed fractions to both pattern 1 and pattern 2. The clinical dataset comprised 30 treatment fractions from 28 patients.

S/I: superior–inferior; A/P: anterior–posterior; L/R: left–right.
