# Supplementary material for: Dosimetric impact of baseline shift plan in a magnetic resonance linear accelerator according to target motion and plan characteristics
Source: J Appl Clin Med Phys. 2026 Oct 20;27(11):e70861. doi: 10.1002/acm2.70861 (PMC13682616; doi:10.1002/acm2.70861)
Supplement: Supplementary file 5 [file ACM2-27-e70861-s005.docx]

**Supplementary Table S4. Spearman's correlation coefficients between isocenter-to-target-centroid distance and target coverage (D98%) for BLS plans.**

| Shift direction | Shift magnitude (mm) | Prostate | | Liver | | Pancreas | |
| --- | --- | --- | --- | --- | --- | --- | --- |
|  |  | Spearman's correlation coefficient | p value | Spearman's correlation coefficient | p value | Spearman's correlation coefficient | p value |
| Superior–inferior shift | 1 | -0.721 | **0.024** | 0.091 | 0.811 | 0.115 | 0.759 |
|  | 3 | -0.745 | **0.018** | -0.188 | 0.608 | 0.333 | 0.349 |
|  | 5 | -0.552 | 0.104 | 0.406 | 0.247 | 0.261 | 0.470 |
|  | 10 | -0.176 | 0.632 | 0.079 | 0.838 | 0.212 | 0.560 |
| Posterior shift | 1 | -0.479 | 0.166 | 0.358 | 0.313 | 0.345 | 0.331 |
|  | 3 | -0.661 | **0.044** | 0.212 | 0.560 | 0.224 | 0.537 |
|  | 5 | -0.648 | **0.049** | 0.455 | 0.191 | 0.103 | 0.785 |
|  | 10 | -0.455 | 0.191 | 0.503 | 0.143 | 0.127 | 0.733 |

D98%: dose received by 98% of the target volume. Target coverage was evaluated using planning target volume (PTV)−rectum−bowel D98% for prostate cancer, PTV D98% for liver cancer, and PTVp−PRVs D98% for pancreatic cancer. Bold values indicate statistically significant correlations (p < 0.05).
