# Supplementary material for: Dosimetric impact of baseline shift plan in a magnetic resonance linear accelerator according to target motion and plan characteristics
Source: J Appl Clin Med Phys. 2026 Oct 20;27(11):e70861. doi: 10.1002/acm2.70861 (PMC13682616; doi:10.1002/acm2.70861)
Supplement: Supplementary file 6 [file ACM2-27-e70861-s006.docx]

**Supplementary Table S2. Relative electron densities assigned to each structure in the simulation dataset.**

| Disease site | Structure | Relative electron density | |
| --- | --- | --- | --- |
|  |  | Mean | SD |
| Prostate | CTV | 1.046 | — |
|  | Rectum | 1.039 | — |
|  | Bladder | 1.025 | — |
|  | Left femoral head | 1.178 | — |
|  | Right femoral head | 1.179 | — |
|  | Bone | 1.205 | — |
|  | Body | 0.992 | — |
| Liver | ITV | 1.041 | 0.071 |
|  | Spinal cord | 1.049 | 0.008 |
|  | Heart | 1.048 | 0.016 |
|  | Left kidney | 1.046 | 0.016 |
|  | Right kidney | 1.043 | 0.017 |
|  | Liver | 1.059 | 0.018 |
|  | Lung | 0.303 | 0.069 |
|  | Bone | 1.159 | 0.025 |
|  | Body | 0.964 | 0.025 |
| Pancreas | ITV | 1.058 | 0.017 |
|  | Duodenum | 1.028 | 0.056 |
|  | Small bowel | 0.981 | 0.089 |
|  | Large bowel | 0.833 | 0.184 |
|  | Stomach | 0.887 | 0.158 |
|  | Spinal cord | 1.046 | 0.004 |
|  | Left kidney | 1.066 | 0.009 |
|  | Right kidney | 1.072 | 0.005 |
|  | Liver | 1.071 | 0.006 |
|  | Lung | 0.354 | 0.068 |
|  | Bone | 1.154 | 0.027 |
|  | Body | 0.967 | 0.034 |

Values are the relative electron densities used for dose calculation in the simulation dataset. For the prostate cases, a fixed value calculated from 20 patients was assigned to all patients; therefore, the inter-patient standard deviation (SD) is not shown. For the liver and pancreatic cases, the values were derived from each patient's planning CT and are presented as the mean ± SD across 10 patients. The same assignment method was used for the clinical dataset. CTV: clinical target volume; ITV: internal target volume.
